# Identification of V-ATPase as a molecular sensor of SOX11-levels and potential therapeutic target for mantle cell lymphoma

**DOI:** 10.1186/s12885-016-2550-4

**Published:** 2016-07-18

**Authors:** Venera Kuci Emruli, Roger Olsson, Fredrik Ek, Sara Ek

**Affiliations:** Department of Immunotechnology, Lund University, Medicon Village, Scheelevägen 8, 223 87 Lund, Sweden; Department of Experimental Medical Science, Chemical Biology & Therapeutics, Lund University, Lund, Sweden

**Keywords:** V-ATPase, SOX11, Mantle cell lymphoma

## Abstract

**Background:**

Mantle cell lymphoma (MCL) is an aggressive disease with short median survival. Molecularly, MCL is defined by the t(11;14) translocation leading to overexpression of the *CCND1* gene. However, recent data show that the neural transcription factor SOX11 is a disease defining antigen and several involved signaling pathways have been pin-pointed, among others the Wnt/β-catenin pathway that is of importance for proliferation in MCL. Therefore, we evaluated a compound library focused on the Wnt pathway with the aim of identifying Wnt-related targets that regulate growth and survival in MCL, with particular focus on SOX11-dependent growth regulation.

**Methods:**

An inducible SOX11 knock-down system was used to functionally screen a library of compounds (*n* = 75) targeting the Wnt signaling pathway. A functionally interesting target, vacuolar-type H^+^-ATPase (V-ATPase), was further evaluated by western blot, siRNA-mediated gene silencing, immunofluorescence, and flow cytometry.

**Results:**

We show that 15 out of 75 compounds targeting the Wnt pathway reduce proliferation in all three MCL cell lines tested. Furthermore, three substances targeting two different targets (V-ATPase and Dkk1) showed SOX11-dependent activity. Further validation analyses were focused on V-ATPase and showed that two independent V-ATPase inhibitors (bafilomycin A1 and concanamycin A) are sensitive to SOX11 levels, causing reduced anti-proliferative response in SOX11 low cells. We further show, using fluorescence imaging and flow cytometry, that V-ATPase is mainly localized to the plasma membrane in primary and MCL cell lines.

**Conclusions:**

We show that SOX11 status affect V-ATPase dependent pathways, and thus may be involved in regulating pH in intracellular and extracellular compartments. The plasma membrane localization of V-ATPase indicates that pH regulation of the immediate extracellular compartment may be of importance for receptor functionality and potentially invasiveness in vivo*.*

**Electronic supplementary material:**

The online version of this article (doi:10.1186/s12885-016-2550-4) contains supplementary material, which is available to authorized users.

## Background

Mantle cell lymphoma (MCL) is an aggressive subtype of B cell lymphoma, with only 5 year median survival [1]. The disease is defined by the t(11;14) translocation, resulting in overexpression of the *CCND1* gene and subsequent promotion of G1 to S cell cycle transition. Additional specific disease marker include the neural transcription factor, SOX11, overexpressed in >95 % of MCL cases [2, 3]. Despite recent major therapeutic progress, relapses are common and long-term survival remains poor, and novel targets with curative potential are sought among pathways important for MCL cell survival.

Functionally, MCL is characterized by a number of different genetic aberrations [4], and efforts have focused on targeting the constitutive NFĸB signaling [5], BTK [6] but also Wnt signaling. Wnt signaling is of vital importance both for promotion of lymphomagenesis in MCL [7], but also for survival and evolution, as assessed by gene expression analysis [8, 9]. In a previous siRNA screen, we identified the Wnt receptor FZD2 to be functionally active and affecting proliferation in MCL [10]. Wnt is of importance in a wide variety of tumors and may be specifically interesting for development of therapies that target cancer stem cells [11], with limited off-target effects [12]. This potential has recently been demonstrated in MCL where Wnt-targeting substances were particularly effective in eradication of lymphoma-initiating cells [13].

We and others have shown that the neural transcriptional factor SOX11 is a highly specific diagnostic [2], functional [14–16], and prognostic antigen [17]. SOX11 has been shown to act through a number of signaling pathways, including TGF-β signaling [14], plasmacytic differentiation [18], angiogenesis [19], but also Wnt [20]. Homologous transcription factors to SOX11, the SOX C family, have also been shown to interact with Wnt [21].

With the aim to identify novel targets for therapy in MCL through search at the intersection of SOX11/Wnt signaling, we performed a compound evaluation of an annotated library with 75 compounds interacting either as inhibitors or activators of the Wnt-signaling pathway, and investigated (i) the stand-alone effect and (ii) the SOX11-dependent effect on proliferation in MCL cells.

Results showed that among the evaluated 75 substances affecting Wnt-signaling, 15 compounds resulted in reduced proliferation in all the three different MCL cell lines evaluated. Further, upon filtering for differential response in relation to SOX11 level, three substances directed to two different targets (V-ATPase and Dkk1) were identified. Further validation studies were focused on substances targeting V-ATPase, and confirmed that both the V-ATPase specific inhibitors bafilomycin A1 and the analogue concanamycin A result in SOX11-dependent growth reduction. V-ATPase is a known regulator of intra- and extracellular pH, thus normal expression of this proton pump is of critical point for maintenance of ideal cellular pH [22].

In this study, we show for the first time that V-ATPase inhibitors effectively reduce proliferation in MCL cells, are sensitive to SOX11 status and that V-ATPase is expressed on the surface of both primary MCL cells and cell lines, and thus an interesting therapeutic target.

## Methods

### Cultivation of cell lines

Three MCL cell lines, Z138, GRANTA-519 and JEKO-1, transfected with an inducible shRNA-vector were used to knock-down SOX11 through addition of 1 μg/ml doxycycline (Sigma-Aldrich, Saint Louis, MO, USA). Briefly, cell lines were maintained as previously described [23] in tet-free R10 medium (RPMI-1640 (Life Technologies, Grand Island, NY, USA) supplemented with 10 % tet-approved fetal bovine serum (Life Technologies) and 20 μM L-glutamine (Life Technologies)), and cultured under standard conditions (humidified atmosphere, 5 % CO_2_, 37 °C). SOX11 protein expression was monitored over time by flow cytometry analysis, performed as previously described [24]. Doxycycline was used to induce down-regulation of SOX11. Thus, SOX11 high cells are referred to as non-induced (SOX11^IND-^) and SOX11 low cells as induced (SOX11^IND+^). SOX11^IND-^ cells express similar SOX11 level compared to non-transfected, wild-type cells. All cell cultures were kept in log phase, at a density of 0.8–2 × 10^6^ cells/ml.

### Molecular substances and reagents

Wnt pathway small molecule library was purchased from Enzo Life Sciences (BML-2838), dissolved in DMSO (10 mM) and stored at −80 °C. Upon treatment of cells, the small molecules were resuspended and diluted in tet-free R10 medium and used immediately, or stored at +8 °C and consumed within a week. Individual compounds bafilomycin A1 (ALX-380-063-M001) and concanamycin A (ALX-380-034-C025) were purchased from Enzo Life Sciences (Farmingdale, NY, USA) as dry powders and dissolved in DMSO.

### Assessment of proliferation using thymidine incorporation

**Fig. 1:**
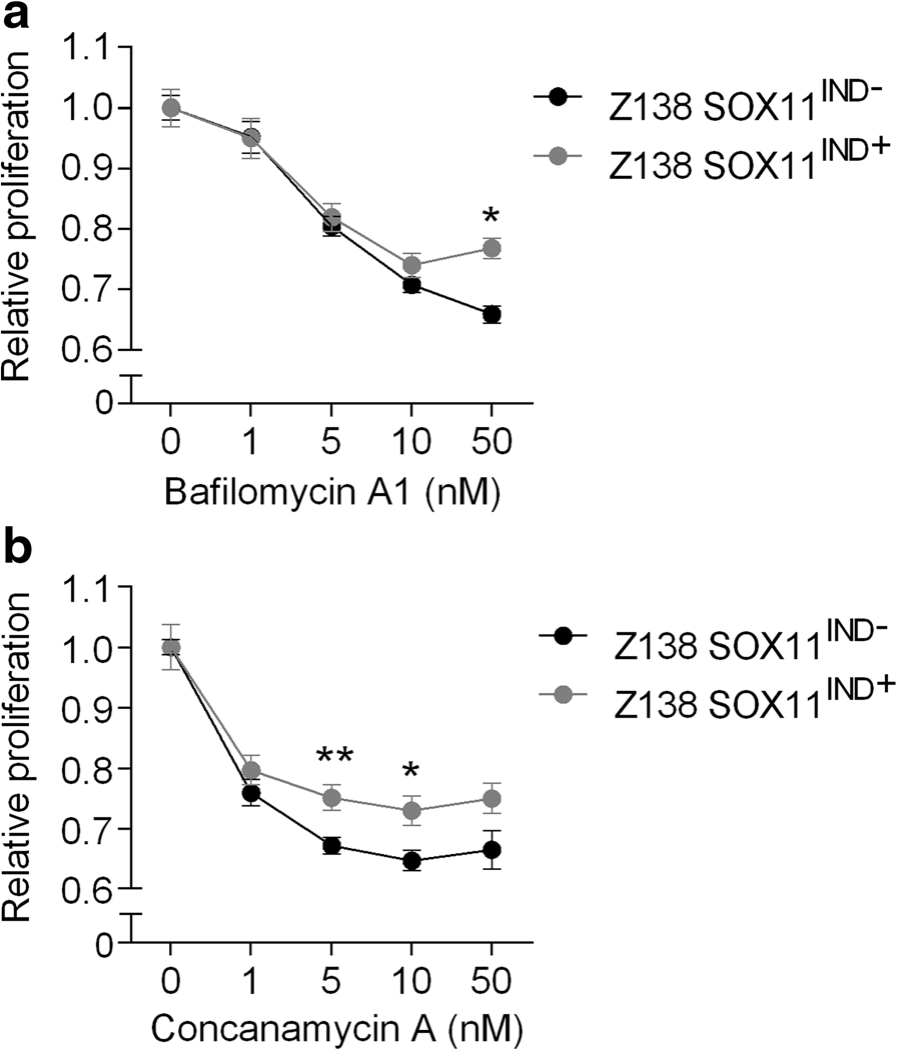
V-ATPase is sensitive to SOX11 status. Assessment of cell proliferation in stably transfected Z138 cells treated with different concentrations of (**a**) bafilomycin A1, and (**b**) concanamycin A, for 24 h. Mean values (*n* = 3 biological replicates per group) are normalized against corresponding non-treated control samples, and error bars indicate SEM. The significance was determined by Student’s *t*-test (*P <0.05, **P <0.005)

**Fig. 2:**
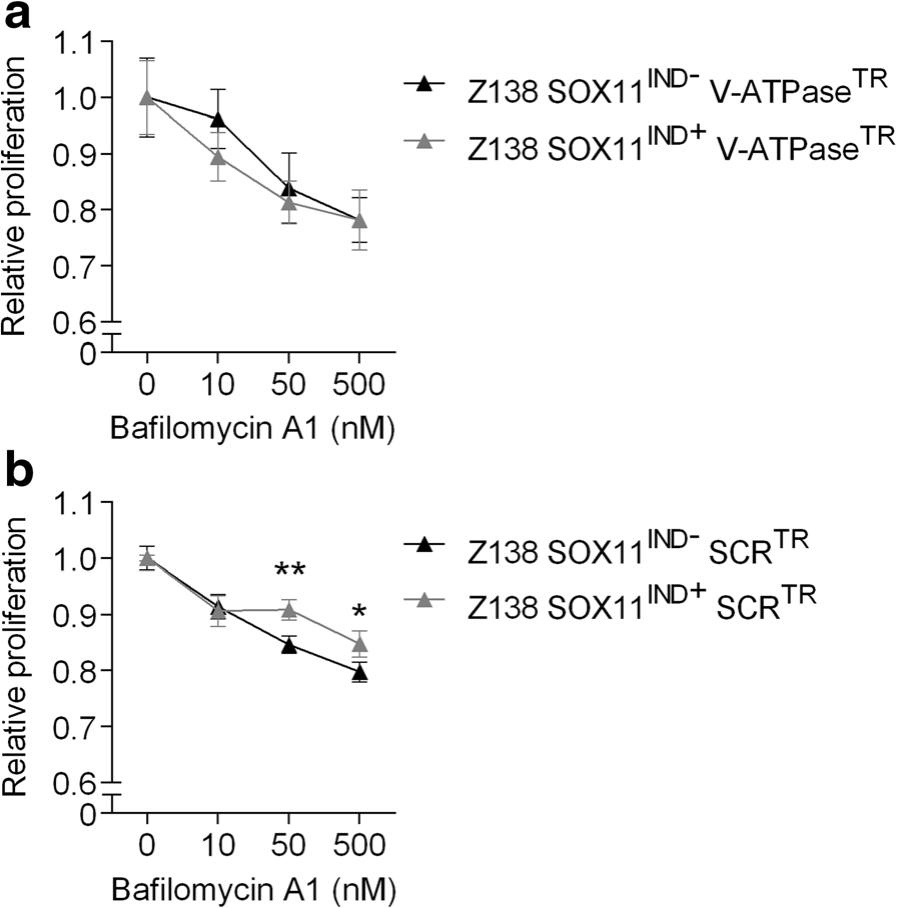
Wild-type V-ATPase level is a pre-requisite for SOX11-dependent bafilomycin A1-induced growth inhibition. Cell lines with altered expression of SOX11 (high/SOX11^IND-^, low/SOX11^IND+^) were transfected with (**a**) a pool of siRNAs targeting V-ATPase or (**b**) a control siRNA (SCR), and treated with different concentrations of bafilomycin A1 for up to 48 h. The data here represents the 48 h time-point. **a** Following treatment with bafilomycin A1, cells with knocked V-ATPase fail to show SOX11-dependent bafilomycin A1-induced growth inhibition. **b** Cells with functional V-ATPase show SOX11-dependent, bafilomycin A1-induced growth reduction at 50 and 500 nM. Thus, we conclude that the SOX11-dependent, V-ATPase inhibitor-induced growth inhibition is related to the function of V-ATPase. Mean values are normalized against corresponding non-treated control samples, and error bars indicate SEM. The significance was determined by Student’s *t*-test (*P <0.05, **P <0.005). V-ATPase^TR^ refers to transient knock-down of V-ATPase, and SCR^TR^ refers to scrambled control used in transient knock-down experiments

Fifty thousand cells per well were plated in 96 well Cytostar-T plates (PerkinElmer, Waltham, MA, USA) and cultured for 24 h prior to treatment with a library of compounds interacting with the Wnt pathway (*n* = 75), at concentrations of 0.5, 2, 5 and 10 μM. Cell proliferation was evaluated 0, 24, 48 and 72 h after addition of small molecules, by measuring incorporation of [14C]-thymidine (PerkinElmer) using a Wallac 1450 MicroBeta liquid scintillation counter (PerkinElmer). The relative proliferation is presented as relative to the non-treated control, at the specific time-point. Each data-point is represented by a minimum of three replicates (Figs. 1 and 2).

### Cell viability measurements using Cell Titer Glow

Fifty thousand cells per well were plated out in 96 well assay plates and cultured for 24 h before treatment with Wnt-targeting small molecule library (*n* = 75) for up to 48 h. On the day of measurement, cells were equilibrated for 30 min at room temperature, treated with CellTiter-Glo reagent (Promega, Madison, WI, USA), mixed and allowed to equilibrate for additional 10 min, before the luminescent signal was recorded using a FLOUstar Omega (BMG Labtech, Ortenberg, Germany) instrument.

### Transient knock-down of V-ATPase

To obtain co-knock of V-ATPase and SOX11, Z138 SOX11^IND+^/SOX11^IND-^ cells were transfected using an Amaxa 4D-Nucleofector (Lonza, Basel, Switzerland). Cells, 2.5 million were resuspended in SF solution (Lonza) and mixed with 500 nM pool of siRNA (Sigma-Aldrich) targeting V-ATPase. In each reaction a scrambled sequence (SCR) was used as a control.

After transfection, 50,000 cells were plated out in 96 well Cytostar-T plates, and grown for 24 h, before treated with different concentrations of bafilomycin A1 and concanamycin A (Enzo Life Sciences), respectively, for up to 48 h. Cell proliferation, by incorporation of [14C]-thymidine, was measured as described above.

### Immunofluorescence

Localization of V-ATPase on primary MCL cells and cell lines was evaluated using immunofluorescence. Briefly, 50,000 cells were centrifuged onto glass slides using a cytospin (Shandon, Pittsburgh, USA) and fixed in 4 % paraformaldehyde for 10 min. The cells were rinsed with PBS (Life Technologies) and blocked with 5 % FBS, 5 % goat serum (Life Technologies) in PBS for 30 min. Unless otherwise indicated, all incubations were carried out at room temperature. For the primary staining, cells were incubated with a rabbit antibody targeting the V0a1 domain of V-ATPase (ATP6V0A1) (sc-28801, Santa Cruz Biotechnology, Dallas, TX, USA) overnight, and then washed with PBS. The cells were then incubated with a secondary antibody, a goat anti-rabbit IgG-Alexa 488 (Life Technologies), for 1 h. MCL cells derived from patient samples were stained also for CD20 expression. Briefly, these cells were incubated for 1 h with a primary mouse anti-CD20 antibody (Dako) followed by an equally long incubation with a secondary goat anti-mouse IgG-Alexa 568 (Life Technologies). Finally, cell nuclei were stained with 300 nM DAPI (Life Technologies) and the slides were mounted with ProLong Gold anti-fade mounting medium (Life Technologies). Fluorescence images were visualized using a fluorescence Nikon ECLIPSE 80i microscope (Nikon Instrument Inc., NY, USA) equipped with a 40x objective (Nikon Plan Fluor 40x/0.75 DIC M/N2, Nikon Instrument Inc, Melville, NY). Images were captured using a Nikon DS-U2/L2 USB camera (Nikon Instrument Inc, Melville, NY) equipped with DAPI, FITC, and Texas Red filters. The exposure times used were 10 ms DAPI, 100 ms FITC, and 400 ms Texas Red.

### Flow cytometry

Cell surface expression of V-ATPase was assessed on six different MCL cell lines and two primary samples, using flow cytometry. In total, two different antibodies targeting ATP6V0A1 were used (sc-28801, Santa Cruz Biotechnology; PA5-25033, ThermoFisher, Waltham, MA, USA). Briefly, 10^5^ cells of interest were washed and stained with anti-ATP6V0A1 for 30 min at 4 °C. After washing with PBS, the cells were stained with a secondary antibody, goat anti-rabbit IgG-FITC (BD Pharmingen) for additional 30 min at 4 °C. Isotype controls were included. Stained cells were washed prior to analysis using FACS Canto II flow cytometer (BD Bioscience). Data analysis was performed using the software FCS Express 4 Flow Research Edition (De Novo Software, Los Angeles, CA USA).

### Statistics

The graphs were created using GraphPad Prism v.6.05 (GraphPad Software, La Jolla, CA, USA). Student’s *t*-test was utilized in all analyses, unless otherwise indicated. Significance in the figures is reported as *P <0.05, **P <0.005.

## Results

### Screening of small molecules targeting Wnt signaling identify V-ATPase as sensitive to SOX11 levels

A library of 75 small molecule compounds targeting proteins of the Wnt signaling pathway (Additional file 1: Table S1), were functionally screened in three MCL cell lines at different concentrations. Cell proliferation was assessed in all three cell lines, and viability in one cell line (Z138). Fifteen substances showed >20 % reduced proliferation (compared to DMSO controls) in the three different cell lines at minimum of two different concentrations per cell line (Additional file 1: Table S2). Involved targets include Dkk1, GSK-3b, TCf4/ β-catenin, CK1a, LRO5/6 and V-ATPase. In addition, seven of these substances also showed a reduced viability (>20 %) upon treatment of Z138 cells (Additional file 1: Table S3). Next, filtering (cutoff: SOX11^IND+^/SOX11^IND-^ >1.5 in minimum 5 out of 10 proliferation experiments) for differential response to SOX11 level was performed, and three substances targeting two different targets were identified (Table 1). V-ATPase, a specific inhibitor of vacuolar-type H+−ATPase, was selected for further validation and functional analysis.

**Table 1:** Substances with SOX11-dependent inhibition of proliferation

Drug	Target	Mean SOX11^IND+^/SOX11^IND-^
bafilomycin A1	V-ATPase	6.6
trichostatin A	Dkk1	25.1
5-Aza-2-deoxycytidine (decitabine)	Dkk1	2.5

Mean ratios of SOX11^IND+^/SOX11^IND-^ calculated from GRANTA-519, JEKO-1 and Z138

### Cellular SOX11 protein level affects treatment response to V-ATPase inhibitors

All validation studies were performed using SOX11 high and low Z138 MCL cell line. Cells were treated with different concentrations of bafilomycin A1, and proliferation was measured 0, 24, 48 and 72 h after treatment. Already after 24 h, SOX11 high cells (SOX11^IND-^) were shown to be more sensitive than SOX11 low cells (SOX11^IND+^), when treated with ≥10 nM bafilomycin A1 (Fig. 1a). The difference in treatment response was however, only significant at 50 nM. The narrow concentration range for specific bafilomycin A1 induced V-ATPase inhibition has also been seen in previous studies [25, 26]. Using a second V-ATPase inhibitor, concanamycin A, we could confirm the V-ATPase specific, SOX11-dependent growth reduction (Fig. 1b). Also concanamycin A show a narrow concentration range for SOX11-dependent growth inhibition, although at lower concentrations (5 and 10 nM). In accordance to previous studies on V-ATPase inhibition, concanamycin A showed a stronger potency compared to bafilomycin A1 [27, 28].

### siRNA mediated knock-down of V-ATPase abolish the difference in bafilomycin A1 sensitivity between SOX11 high and low cells

To further validate that SOX11-dependent growth inhibition induced by bafilomycin A1 and concanamycin A is related to V-ATPase activity, we investigated if the sensitivity to bafilomycin A1 is altered upon transient knock-down of V-ATPase. Cells with modified expression of SOX11 were transfected with siRNA targeting V-ATPase or a SCR control. Cells with reduced V-ATPase level showed no SOX11-dependent, bafilomycin A1 induced growth inhibition (Fig. 2a). As expected, control experiment (using scrambled control) demonstrate SOX11-dependent bafilomycin A1 induced growth reduction (Fig. 2b). Thus, SOX11-dependent sensitivity to V-ATPase inhibitors is dependent on wild-type V-ATPase levels.

### Cellular protein levels of V-ATPase and SOX11 are not co-regulated

The difference observed between SOX11 high and low cells in response to treatment with bafilomycin A1 and concanamycin A, respectively, made us investigate the relationship betweeen the target protein, V-ATPase, and SOX11. Gene expression data (Additional file 2: Figure S1a) vagely pointed towards an anti-correlation between V-ATPase and SOX11. However, this could not be confirmed using RT-PCR (data not shown), western blot analysis (Additional file 2: Figure S1b) or immunofluoresence (data not shown). Thus, the difference in response to V-ATPase inhibitors can not be explained by a difference in V-ATPase protein level in SOX11 high/low cells.

### Immunofluoresence and flow cytometry analysis show that V-ATPase are found at the plasma membrane of cells

**Fig. 3:**
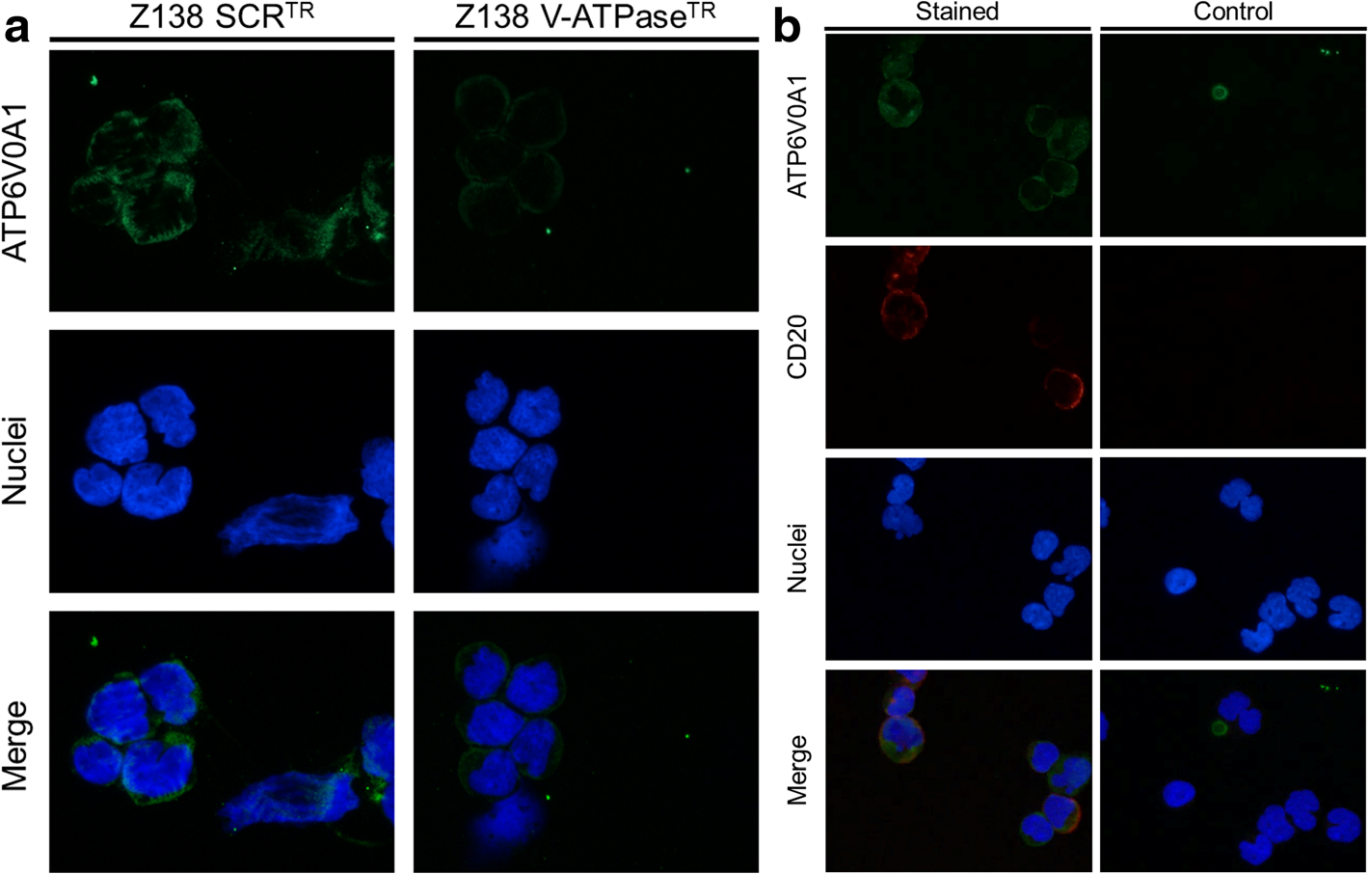
V-ATPase is localized at the plasma cell membrane in primary and MCL cell lines. Localization of V-ATPase in (**a**) Z138 control cells (SCR^TR^), transiently V-ATPase knocked Z138 cells (V-ATPase^TR^), and (**b**) primary MCL cells. Cells were centrifuged on glass slides and stained for V-ATPase. The nuclei were counterstained by DAPI. MCL primary cells were also stained for CD20 to visualize tumor cells. The MCL control sample was not treated with any primary antibody. All staining’s were analyzed using a Nikon ECLIPSE 80i fluorescence microscope

V-ATPases are known to be expressed in the membrane of intracellular organells, but also at the plasma membrane of certain cells [29]. Plasma membrane localization of V-ATPase has been reported as functionally important for tumor invasiveness and metastasis in a number of tumors [30, 31]. Thus, to investigate the localization of V-ATPase in both MCL primary cells and cell lines, we performed immunofluoresence staining by using a commercially available antibody targeting the membrane associated domain V0a1, ATP6V0A1 (Fig. 3). In both cases, expression of V-ATPase was shown to be located at, but not limited to, the plasma membrane.

**Fig. 4:**
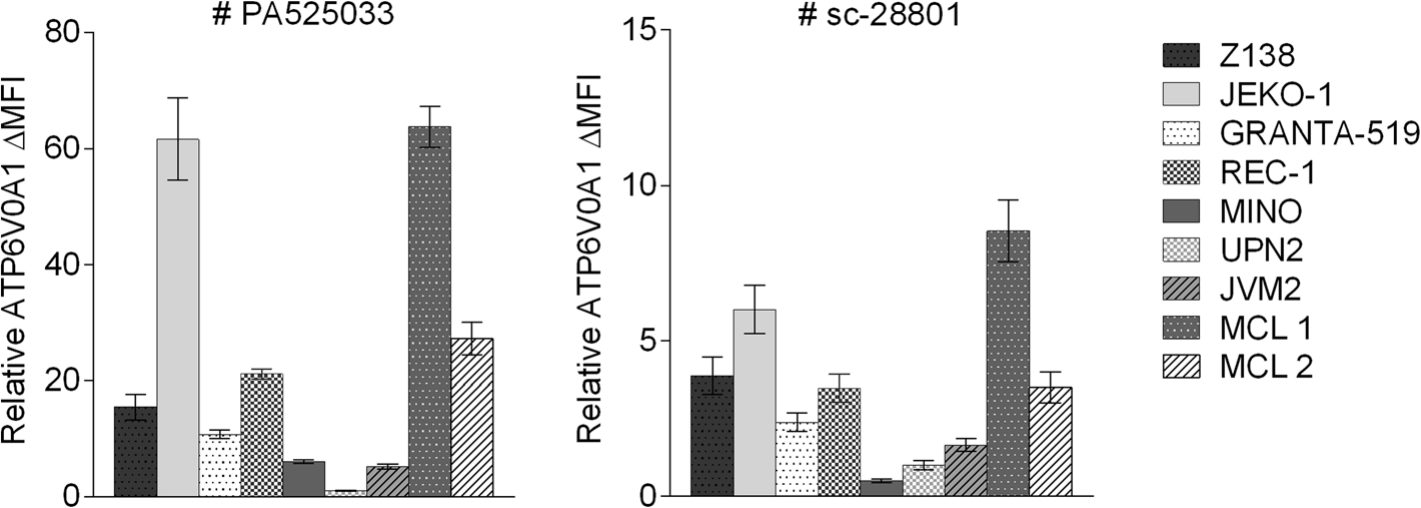
Assessment of V-ATPase cell surface expression using flow cytometry analysis. For each cell line, a ΔMFI was calculated as the difference between the mean fluorescence intensity (MFI) of ATP6V0A1 staining compared to unstained control, (MFI_ATP6V0A1_ – MFI_control_). The relative ATP6V0A1 expression was calculated by scaling all data to the cell line with the lowest ΔMFI for #PA525030 (UPN2). Error bars indicate SEM of three technical replicates

To further confirm the cell surface expression of V-ATPase and investigate if the protein is accesible for specific antibodies, flow cytometry analyses were performed using both primary MCL samples (*n* = 2) and cell lines (*n* = 7). Two commercially available antibodies targeting the a1 subunit of the V0 domain were used. In general, lower signal intesities were acquired for the sc-28801 compared to the PA525033 V0a1 specific V-ATPase targeting antibody. However, similar variation in V-ATPase levels across the different samples were detected for both antibodies (Fig. 4). JEKO-1 and MCL patient sample 1 showed highest level of ATP6V0A1 among the analyzed samples, whereas UPN2, MINO and JVM2 showed a limited surface expression of this subunit.

In summary, using a Wnt-specific molecular library, we identified bafilomycin A1 as a potent SOX11-dependent inhibitor of growth in MCL cells. The SOX11-dependent effect of V-ATPase inhibitors (bafilomycin A1 and concanamycin A) was dependent on wild-type levels of V-ATPase, as assessed by knock-down experiments. We further show that V-ATPase is located at the plasma membrane in MCL cells, and thus of potential importance for tumor metastasis and invasiveness in these tumors.

## Discussion

Wnt signaling has a profound effect on MCL [7–9] and a recent study points to a connection with SOX11, a disease-defining antigen in MCL [20]. With the aim of identifying novel targets for therapeutic intervention of MCL, we have searched for targets at the cross-section of Wnt and SOX11 signaling.

To identify Wnt-related small molecules with anti-proliferative effect, and specifically responsive to SOX11 levels, a compound evaluation using an annotated library targeting the Wnt-pathway was performed. Within the Wnt-pathway targeting library, 15 out of 75 molecular compounds had the capacity to reduce proliferation in three different MCL cell lines. Functional targets include Dkk1, GSK-3b, TCF4/ β-catenin, CK1a, LRO5/6 and V-ATPase. Further, by comparing the effect in SOX11 high and low cells, three substances with SOX11-dependent effect on proliferation were identified (aza-2 deoxycytidine/Dkk1, trichostatin A/Dkk1 and bafilomycin A1/V-ATPase). Further validation and characterization was focused on V-ATPase as a potential target using both bafilomycin A1 and an analogue, concanamycin A, as specific V-ATPase targeting substances. The potential of bafilomycin A1 to target V-ATPase and suppress proliferation was described by Tashiro and colleagues in 1993 [32] and later, further characterization of the families of bafilomycins and concanamycins as specific V-ATPase inhibitors was performed [33].

Experiments show that both bafilomycin A1 and concanamycin A exert anti-proliferative effect and induce cell death in MCL cell lines. A significant difference in response between SOX11 high and low expressing cell lines is seen at concentrations (ca 10–50 nM) where bafilomycin A1 and concanamycin A are reported to be specific for V-ATPase, with no off-target effect [25, 26]. Using siRNA-mediated knock-down of V-ATPase, the SOX11-dependent sensitivity to bafilomycin A1 inhibition was also shown to be dependent on normal V-ATPase levels, thus we conclude that the effect is specifically related to V-ATPase function.

V-ATPase is an ATP-driven enzyme that transforms the energy of ATP hydrolysis to electrochemical potential difference of protons across diverse biological membranes via the primary active transport of H^+^ [22]. Thus, V-ATPase has a major function as regulator of intra- and extracellular pH, and normal expression of this proton pump is of critical point for maintenance of ideal cellular pH. Overexpression of V-ATPase has been reported in several tumors where it has shown to promote acidification of the extracellular environment, and thus favor tumor growth [34, 35] invasiveness [36, 37] and resistance to cytotoxic agents [38, 39]. The function of V-ATPase has recently been reviewed more extensively elsewhere [29]. It has further been shown that Wnt signaling is dependent on acidification and thus, functional V-ATPase that can mediate proton fluxes contribute to normal Fz receptor function and successful Wnt signaling [40, 41].

In order to exclude that V-ATPase levels are different in SOX11 high and low cells, the level of V-ATPase was studied using gene expression analysis, western blot (WB) and immunofluorescence. Upon gene expression mining, correlation analysis indicated that SOX11 and V-ATPase might be anti-regulated, but this could not be confirmed with either RT-PCR or western blot analysis. Visual examination of fluorescence imaging of individual cells, could not either detect any difference. Thus, we conclude that difference in V-ATPase inhibition upon SOX11 regulation is not related to a differential level of V-ATPase protein in SOX11 high versus low cells.

It has been reported that a number of different tumor cells express V-ATPase at the plasma membrane [30]. The activity of V-ATPase at the plasma membrane has been linked to metastatic potential, suggesting that V-ATPase provides acidic extracellular environment necessary for invasion. We used antibodies targeting the V0a1 domain to investigate if the V-ATPase complex was present in the plasma membrane. Fluoresence microscopy image analysis showed that V-ATPase is clearly present in the plasma membrane, but likely also to a lesser extent in other intracellular membranes. Flow cytometry analysis was used to confirm that the V0a1 domain is accessible at the surface of MCL cells, and thus potentially able to be targeted by antibodies with therapeutic potential.

Functionally, V-ATPase has been pin-pointed as an interesting target to reduce metastatic disease [31, 42, 43], but it is also well known that the target is toxic when treated with small molecular inhibitors, with systemic effect. It has been shown that the activity of V-ATPase localized in the plasma membrane is critical for invasion of breast cancer cells [44] and specific isoforms have been shown to target V-ATPase to the plasma membranes [45, 46]. Thus, the availability of the protein on the cell surface of tumor cells may enable antibody-based specific therapy, and would constitute a less toxic way to target V-ATPase at the surface of tumor cells without disrupting function in intracellular membrane of normal cells, as penetration of the plasma membrane by antibodies are considered to be minimal.

Disregarding toxicity, inhibition of V-ATPase has shown great potential to sensitize tumor cells to various cytotoxic agents by disrupting the pH gradient between the cell cytoplasm and lysosomal compartment. For example, in multidrug-resistant renal epithelial cells, both bafilomycin A1 and concanamycin A were shown to reverse the resistance to anthracyclines [47]. Similarly, You and colleagues [48] showed that inhibition of V-ATPase by siRNA recovered sensitivity to chemotherapy in breast cancer cells. Moreover, in B cell lymphoma cells with reduced expression of CD20, induced by bortezomib treatment, inhibition of V-ATPase led to restorage of CD20 levels, enabling further treatment with rituximab [49].

V-ATPase is also well known to be involved in cancer metastasis and tumor progression. Already in late 90′s, Ohta and colleagues showed that V-ATPase overexpression is characteristic for invasive pancreatic tumors and suggested that V-ATPase may play a crucial role in tumor progression [50]. Similarly, Sennoune and colleagues [31] showed that highly metastatic and invasive breast tumor cells exhibit a significantly higher plasma membrane V-ATPase activity compared to less invasive breast tumor cells. Other studies, involving leukemic stem cells [51], esophageal squamous [39] and non-small-cell lung cancer [52], show a direct relationship between V-ATPase expression and enhanced drug resistance.

## Conclusions

In conclusion, we provide novel information that V-ATPase is sensitive to SOX11 levels, indicating that SOX11 affect cellular pH regulation. V-ATPase is localized on the membrane of MCL cells, and may thus be involved in metastasis and is an interesting candidate for antibody-based treatment.

## Abbreviations

MCL, mantle cell lymphoma; V-ATPase, vacuolar-type H+−ATPase

## Additional files

Additional file 1: Table S1. Library of 75 small molecule inhibitors targeting proteins of the Wnt signaling pathway. **Table S2**. Small molecule inhibitors with an anti-proliferative effect observed in three different MCL cell lines at a minimum of two different concentrations per cell line. Reduction in proliferation is presented as mean percentage compared to the used vehicle control, DMSO. **Table S3**. Small molecule inhibitors with an anti-viability effect on Z138 cells. Reduction in viability is presented as mean percentage compared to the used vehicle control, DMSO. (DOCX 28 kb) Additional file 2: Additional methods including gene expression studies and western blotting. **Figure S1**. The expression of isoform a1 of V-ATPase on a) mRNA is partly anti-correlated to SOX11, as observed here in two different cell line models with altered expression of SOX11. However, no correlation of V-ATPase to SOX11 is visible on b) protein level. The mRNA expression of V-ATPase was assessed by HuGene ST 1.0 arrays. Each data point represents a unique sample. GAPDH expression was used as a protein-loading control for western blot analysis. (DOCX 111 kb)
