# Supplementary material for: Identification of V-ATPase as a molecular sensor of SOX11-levels and potential therapeutic target for mantle cell lymphoma
Source: BMC Cancer. 2016 Jul 18;16:493. doi: 10.1186/s12885-016-2550-4 (PMC4949756; doi:10.1186/s12885-016-2550-4)
Supplement: Additional file 1: Table S1. — Library of 75 small molecule inhibitors targeting proteins of the Wnt signaling pathway. Table S2. Small molecule inhibitors with an anti-proliferative effect observed in three different MCL cell lines at a minimum of two different concentrations per cell line. Reduction in proliferation is presented as mean percentage compared to the used vehicle control, DMSO. Table S3. Small molecule inhibitors with an anti-viability effect on Z138 cells. Reduction in viability is presented as mean percentage compared to the used vehicle control, DMSO. (DOCX 28 kb) [file 12885_2016_2550_MOESM1_ESM.docx]

**Additional file 1**

Table S1**.** Library of 75 small molecule inhibitors targeting proteins of the Wnt signaling pathway.

| **Name** | **Target** |
| --- | --- |
| Foxy-5 | Wnt5a |
| Anandamide | Wnt5a |
| QS-11 | ARFGAP1 |
| Diarylsulfonesulfonamide | sFRP-1 |
| IQ1 | PP2A |
| SB-216763 | GSK-3b |
| BIO | GSK-3b |
| TWS-119 | GSK-3b |
| CHIR99021 | GSK-3b |
| IM-12 | GSK-3b |
| AR-A014418 | GSK-3b |
| Kenpaullone | GSK-3b |
| Sodium valproate | GSK-3b |
| BML-284 | GSK-3b independent |
| Deoxycholic acid | β-catenin |
| Resveratrol | Ak t& Erk / GSK-3b |
| PGE2 | cAMP/PKA |
| WAY-262611 | Dkk |
| Purpurogallin | Dkk1 |
| Exifone | Dkk1 |
| Gallic acid | Dkk1 |
| Riluzole | GRM1 |
| LY456236·HCl | GRM1 |
| (-)-Terreic acid | BTK |
| Forskolin | Adenylyl cyclase |
| GW9662 | PPARγ |
| Box5 | Wnt5a |
| TNP-470 | Wnt PCP pathway |
| IWR-1 | Axin |
| XAV939 | Axin / tankyrase |
| IWP-2 | Porcupine |
| Bafilomycin A1 | V-ATPase |
| Sulindac Sulfide | COX-2 |
| Celecoxib | COX-2 |
| Diclofenac·Na | COX-2 |
| Niclosamide | Frizzled / Dishevelled |
| ICG-001 | CREB-binding protein |
| Harmine·HCl | Wnt-dependent gene expression |
| Curcumin | TCf4/β-catenin interaction |
| PNU-74654 | TCf4/β-catenin interaction |
| Quercetin | TCf4/β-catenin interaction |
| BML285 (Diaminoquinazoline) | TCf4/β-catenin interaction |
| NO-ASA | TCf4/β-catenin interaction |
| FH-535 | TCf4/β-catenin interaction |
| Val-Val-Val | Dishevelled/PDZ protein interaction |
| BML286 (3289-8625) | Dishevelled/PDZ domain |
| Sulindac | Dishevelled/PDZ protein interaction |
| EGCG | HBP1 transcriptional repressor |
| Imatinib mesylate | β-catenin signaling |
| Troglitazone | PPARγ |
| Rosiglitazone maleate | PPARγ |
| Cardamonin | Down-regulation β-catenin |
| CCT036477 | β-catenin transcription |
| Bosutinib (SKI-606) | c-Src-dependent β-catenin phosphorylation |
| Carnosol | β-catenin phosphorylation |
| Flavanone | Transcription of β-catenin/Tcf responsive genes |
| Retinoic Acid | Transcription of β-catenin/Tcf responsive genes |
| Pterostilbene | β-catenin and cyclin D levels |
| Hexachlorophene | Siah-1 / Cyclin d |
| DHA | β-catenin degradation |
| EPA | β-catenin degradation |
| JS-K | β-catenin |
| Pyrvinium pamoate | CK1a |
| Apigenin | CK2 |
| Ellagic Acid dihydrate | CK2 |
| D4476 | CK1δ/ε |
| Trichostatin A | Dkk1 |
| 5-Aza-2-deoxycytidine(Decitabine) | Dkk1 |
| Thalidomide | Dkk1/Bmp target genes |
| Genistein | Tyrosine kinase |
| PP2 | Src kinase |
| NCI16221 | LRP5/6 |
| Doxorubicin·HCl (3013-0085) | LRP5/6 |
| 3253-5986 | LRP5/6 |
| Usnic acid | LRP5/6 |

Table S2. Small molecule inhibitors with an anti-proliferative effect observed in three different MCL cell lines at a minimum of two different concentrations per cell line. Reduction in proliferation is presented as mean percentage compared to the used vehicle control, DMSO.

|  |  | **(%) mean reduction in proliferation** | | |
| --- | --- | --- | --- | --- |
| **Name** | **Target** | GRANTA-519 | JEKO-1 | Z138 |
| Trichostatin A | Dkk1 | 47.3 | 99.2 | 99.6 |
| 5-Aza-2-deoxycytidine(Decitabine) | Dkk1 | 81.0 | 89.2 | 87.1 |
| Hexachlorophene | Siah-1 / Cyclin d | 54.2 | 85.6 | 44.5 |
| BML-284 | GSK-3b independent | 94.5 | 97.8 | 99.4 |
| (-)-Terreic acid | BTK | 37.7 | 62.1 | 34.1 |
| Bafilomycin A1 | V-ATPase | 51.5 | 86.1 | 96.5 |
| BML285 (Diaminoquinazoline) | TCf4/β-catenin interaction | 44.8 | 60.1 | 52.9 |
| JS-K | β-catenin | 47.1 | 99.7 | 73.2 |
| NO-ASA | TCf4/β-catenin interaction | 39.4 | 44.8 | 12.8 |
| Pyrvinium pamoate | CK1a | 96.8 | 99.8 | 95.6 |
| Doxorubicin·HCl (3013-0085) | LRP5/6 | 99.5 | 98.9 | 99.9 |
| WAY-262611 | Dkk | 50.3 | 77.8 | 68.3 |
| Bosutinib (SKI-606) | c-Src-dependent β-catenin phosphorylation | 30.7 | 77.6 | 61.6 |
| 3253-5986 | LRP5/6 | 66.7 | 99.3 | 81.4 |
| Niclosamide | Frizzled / Dishevelled | 76.7 | 99.7 | 72.6 |

Table S3. Small molecule inhibitors with an anti-viability effect on Z138 cells. Reduction in viability is presented as mean percentage compared to the used vehicle control, DMSO.

| **Name** | **Target** | **(%) mean reduction in viability** |
| --- | --- | --- |
| Trichostatin A | Dkk1 | 97.9 |
| 5-Aza-2-deoxycytidine(Decitabine) | Dkk1 | 27.8 |
| BML-284 | GSK-3b independent | 83.4 |
| Bafilomycin A1 | V-ATPase | 82.7 |
| Pyrvinium pamoate | CK1a | 51.1 |
| Doxorubicin·HCl (3013-0085) | LRP5/6 | 96.7 |
| 3253-5986 | LRP5/6 | 33.1 |
