# Supplementary material for: Identification of V-ATPase as a molecular sensor of SOX11-levels and potential therapeutic target for mantle cell lymphoma
Source: BMC Cancer. 2016 Jul 18;16:493. doi: 10.1186/s12885-016-2550-4 (PMC4949756; doi:10.1186/s12885-016-2550-4)
Supplement: Additional file 2: — Additional methods including gene expression studies and western blotting. Figure S1. The expression of isoform a1 of V-ATPase on a) mRNA is partly anti-correlated to SOX11, as observed here in two different cell line models with altered expression of SOX11. However, no correlation of V-ATPase to SOX11 is visible on b) protein level. The mRNA expression of V-ATPase was assessed by HuGene ST 1.0 arrays. Each data point represents a unique sample. GAPDH expression was used as a protein-loading control for western blot analysis. (DOCX 111 kb) [file 12885_2016_2550_MOESM2_ESM.docx]

**Additional file 2**

Additional methods

*Gene expression studies*

Five million induced and non-induced cells were lysed in Trizol (Life Technologies, Paisley, UK) 0, 24 and 96 hours after induction. Preparation of tRNA, Human Gene 1.0 ST arrays (Affymetrix Inc., Santa Clara, CA, USA) and raw data extraction was performed as previously described [[13-15](#_ENREF_13), [52](#_ENREF_52)]. Quantile normalization using RMA and quality control was performed in the Expression Console 1.0 software (Affymetrix Inc.). Normalized data was imported into Qlucore Omics Explorer 2.3 (Qlucore AB, Lund, Sweden) where correlation-coefficients to SOX11 were calculated for all transcripts. The ATP6V0A1 transcript, with a correlation factor of -0.7, appeared as top anti-correlated to SOX11.

*Western blotting*

Cell lysis and protein concentration, determined by the BCA standard method, was performed as previously described [[13-15](#_ENREF_13)]. For western blot analysis 30 µg protein was used, and the experiment was carried out as described previously [[30](#_ENREF_30)], with addition of a rabbit anti-ATP6V0A1 (sc-28801, Santa Cruz Biotechnology) and a swine anti-rabbit HRP (P0217, Dako, Glostrup, Denmark).

a)





b)


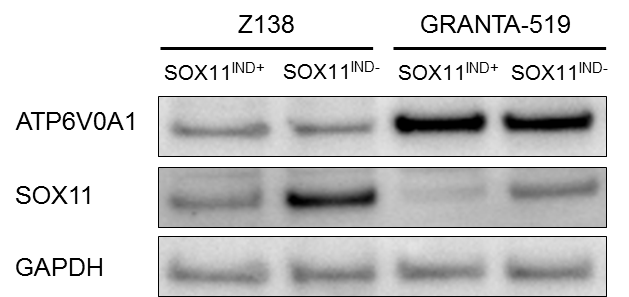


Figure S1. The expression of isoform a1 of V-ATPase on a) mRNA is partly anti-correlated to SOX11, as observed here in two different cell line models with altered expression of SOX11. However, no correlation of V-ATPase to SOX11 is visible on b) protein level. The mRNA expression of V-ATPase was assessed by HuGene ST 1.0 arrays. Each data point represents a unique sample. GAPDH expression was used as a protein-loading control for western blot analysis.
